# Interdisciplinary management of paediatric chronic and complex pain: the experience of a specialised outpatient clinic in Italy

**DOI:** 10.1007/s00431-025-06451-8

**Published:** 2025-09-11

**Authors:** Annalisa Salerno, Francesca Rusalen, Valentina De Tommasi, Mariangela Rosa, Irene Maghini, Grazia Ghiraldo, Franca Benini

**Affiliations:** 1 Department of Women’s and Children’s Health, University of Padua, Padua, Italy; 2 Paediatric Pain and Palliative Care Service, Department of Women’s and Children’s Health, University Hospital of Padua, Padua, Italy

**Keywords:** Paediatric chronic pain, Complex pain, Biopsychosocial model, Multidisciplinary pain management, Outpatient care, Children, Interdisciplinary care

## Abstract

**Purpose:**

This study aimed to describe the structure, patient characteristics, and preliminary clinical outcomes of a dedicated interdisciplinary outpatient clinic for paediatric chronic and complex pain in Italy, with a focus on the feasibility of implementing a biopsychosocial care model.

**Methods:**

We conducted a retrospective review of all patients referred to the Paediatric Specialised Pain Clinic of the University of Padua between January 2023 and May 2024. Data on demographics, clinical diagnoses, pain characteristics, treatments, and follow-up outcomes were collected.

**Results:**

A total of 34 patients (mean age 12.5 years; 56% female) were evaluated over 67 consultations. The most common clinical conditions among patients with pain included musculoskeletal disorders, sickle cell disease, and complex regional pain syndrome. Neuropathic pain accounted for 11% of cases, while the majority (64%) presented with complex pain phenotypes. Treatment plans combined lifestyle interventions, psychological and physical therapies, and—in selected cases—scheduled pharmacological treatments such as gabapentinoids and opioids. A multimodal approach led to ≥ 50% pain reduction in all but one patient lost to follow-up.

*Conclusion*: Our findings support the feasibility and clinical relevance of a structured, interdisciplinary paediatric pain clinic for managing chronic and complex pain. While most paediatric pain can be addressed in non-specialist settings, a subset of patients with complex, disabling pain requires multimodal care within a dedicated interdisciplinary service. This model may represent a scalable prototype for the implementation of paediatric pain services in other regions of Italy and comparable healthcare systems.

**Table:** 

**What is Known:** • *Chronic pain affects more than 20% of children and adolescents. Despite its high prevalence, it is still frequently underrecognized and undertreated, with significant consequences for the overall well-being of the child and family.* *• Interdisciplinary and biopsychosocial approaches are recommended internationally but remain limited in availability.*
**What is New:** • *A structured, multimodal care pathway combining pharmacological and non-pharmacological interventions was feasible even in a regional setting, with ≥ 50% pain reduction in most patients.* •* It is crucial to develop and evaluate new care models specifically designed to address complex pain conditions that remain unresolved within primary or secondary care services.*

## Introduction

Chronic pain affects approximately 21% of children and adolescents [1], significantly impacting their physical, psychological, and social functioning when left unaddressed [2]. Despite its high prevalence, paediatric chronic pain remains frequently underrecognized and undertreated [3]. In some cases, pain management is particularly complex and requires competent and closely monitored interventions. Although most paediatric pain conditions can be managed in primary care or by general paediatricians, a small but significant proportion of patients present with severe, complex, or disabling pain that require the expertise of specialised interdisciplinary teams and access to dedicated care settings [4–6]. In Italy, the management of these children is still fragmented and there are very few centres of reference.

The Paediatric Pain and Palliative Care Centre at the University Hospital of Padua—officially designated as the Regional Referral Centre for the Veneto Region since 2003—was the first of its kind in Italy. It coordinates, supervises, and supports the regional care network in managing children with complex, chronic, or life-limiting conditions, providing interdisciplinary support that extends beyond symptom control to include psychological, social, educational, and bioethical aspects.

Within this framework, a specialised outpatient Paediatric Pain Clinic was formally implemented in 2021 to address the needs of children and adolescents with any type of chronic or complex pain, including those not eligible for palliative care. The clinic offers comprehensive, multimodal, and biopsychosocial care through an interdisciplinary team, aiming to provide individualised treatment plans and ensuring coordinated integration with local health and social services across the region.

The aim of this study was to assess the feasibility of implementing this interdisciplinary care model within a regional healthcare system. We hypothesized that the model could be successfully integrated into routine care, showing adequate patient referral, treatment adherence, effective coordination with community services, and the capacity to manage complex cases in an outpatient setting. Additionally, we report exploratory clinical outcomes—such as reductions in pain intensity and improvements in pain-related functional domains—as preliminary indicators of the model’s adequacy and therapeutic potential.

## Materials and methods

We conducted a retrospective analysis of all patients evaluated at the Paediatric Specialised Pain Clinic of the University of Padua, between January 2023 and May 2024. The Centre provides ongoing professional education in paediatric pain management and ensures 24/7 on-call availability for patients and their families, as well as for healthcare professionals involved in the care of paediatric patients across the Veneto Region (population ~ 4.9 million).

Moreover, it offers both inpatient consultations and outpatient care for children and young adults (up to 23 years) with chronic or complex pain, managed by an interdisciplinary team including a paediatrician specialised in pain medicine, a physiotherapist, a psychologist, and a paediatric nurse.

Patients are typically referred by primary care paediatricians or specialists, usually after unsuccessful first-line treatments such as symptomatic pharmacological therapy (e.g., paracetamol, non-steroidal anti-inflammatory drugs [NSAIDs]), general physiotherapy, and non-specialised psychological support, or due to persistent pain despite specialist evaluations for underlying conditions.

The evaluation and clinical management follow a multimodal, individualised, and biopsychosocial approach, incorporating pharmacological therapies, physical and rehabilitative interventions, psychological support including non-pharmacological techniques such as clinical hypnosis, cognitive behavioural therapy (CBT), eye movement desensitization and reprocessing (EMDR), and relaxation strategies. Additional complementary neuromodulation methods, for example Scrambler Therapy®, are used when appropriate. To ensure continuity, accessibility of care, and long-term engagement, the service relies on the integration of community-based psychological and physiotherapy support. Depending on the type and underlying cause of pain, patients may also be referred to other appropriate medical specialists. Discharge decisions are made on a case-by-case basis, considering sustained clinical improvement, restoration of daily functioning, acquisition of pain management skills, and the possibility to transfer ongoing care to community-based services without the need for close specialist supervision.

Baseline clinical data included: age at the time of the first visit, primary clinical condition, main pain diagnosis, type(s) of treatment administered (pharmacological, rehabilitative, psychological, or combined). Pain conditions were discussed in interdisciplinary meetings and classified according to the predominant pain mechanisms, based on the International Association for the Study of Pain (IASP) conceptual framework (i.e., nociceptive, neuropathic, nociplastic, or mixed mechanisms) [7]. Moreover, we documented when significant psychological contributors were present, reflecting a biopsychosocial interpretation of chronic pain, particularly relevant in paediatric populations, as described in the literature [8]. Feasibility was assessed through a set of selected indicators relevant to the implementation of an interdisciplinary outpatient pain clinic. These included: (1) referral volume, calculated as the total number of new patients evaluated per month; (2) treatment uptake and adherence, assessed through the proportion of patients who initiated and completed the proposed multimodal interventions; (3) coordination with community-based services, evaluated by documenting the percentage of patients referred to local professionals, the types of professionals involved, the presence of shared follow-up, and the time between referral and service activation; and (4) ability to manage patients in an outpatient setting, determined by the absence of hospital admissions or escalation to urgent care, as well as by clinical improvement during follow-up (including a ≥ 50% reduction in pain intensity, decreased use of breakthrough medication, improved sleep and mood, greater participation in daily activities, and reduced school absenteeism).

Descriptive statistics summarised patient characteristics, pain types, treatments, and outcomes.

## Results

From January 2023 to May 2024, a total of 67 consultations were performed, including 25 new patient evaluations (37%), corresponding to a referral volume of approximately 1.5 new patients evaluated per month. A total of 34 patients (mean age 12.5 years, range 2–22; 15 males, 19 females) were evaluated during this period.

### Main clinical presentations and pain diagnosis

Among the 34 patients evaluated, 28 experienced chronic or complex pain (Table 1). Among patients without chronic or complex pain, two presented with insomnia and nocturnal muscle spasms with sleep disturbance, while four had complex medical conditions and care was redirected to specialist palliative services.

**Table 1:** Main clinical presentations and predominant pain mechanisms in patients evaluated at the Paediatric Specialised Pain Clinic

Main clinical presentation (N)	Underlying conditions (*N*)	Predominant pain mechanism
Musculoskeletal disorders (17)	Sickle cell disease without osteonecrosis (2)	Nociceptive/neuropathic
	Sickle cell disease with osteonecrosis (3)	Nociceptive/neuropathic
	Cerebral palsy (3)	Nociceptive (1), nociceptive* (1), nociceptive/neuropathic (1)
	Joint hypermobility (2)	Nociceptive
	Skeletal dysplasia (2)	Nociceptive
	Neuromuscular disease (1)	Nociceptive
	Abdominal surgery (1)	Nociceptive
	Exostosis (1)	Nociceptive/neuropathic
	History of Perthes disease (1)	Nociceptive*
	Coccydynia post-fall trauma (1)	Nociceptive*
Post-traumatic CRPS type 1 (2)		Nociplastic
Phantom limb pain (1)	Post-oncologic surgery	Neuropathic
Neurofibromatosis type 1 (2)	-	Neuropathic
Post-critical illness pain (1)	-	Nociceptive*
Procedural hyperalgesia with anxiety (3)	-	Mixed pain*
Recurrent abdominal pain (1)	-	Nociplastic
Vulvodynia (1)	-	Mixed pain*
Total by predominant pain mechanism(s)		
Nociceptive: 7 (25%), nociceptive/neuropathic: 7 (25%); nociceptive*: 4 (14%), mixed pain* 4 (14%); nociplastic 3 (11%); neuropathic: 3 (11%)		

* with significant psychological contributors. *CRPS* Complex Regional Pain Syndrome

### Treatment and outcomes

With the exception of one patient with CRPS type 1, who did not return after being offered Scrambler Therapy®, all patients (28/29, 97%) adhered to the proposed therapeutic programme, which in 64% of cases (18/28) required the integration of community-based psychological and/or physiotherapy services. Lifestyle changes were the most frequently recommended intervention, applied to all patients with pain and for both patients with sleep disturbances (Table 2).

**Table 2:** Distribution of therapeutic approaches among patients evaluated at the Paediatric Specialised Pain Clinic from January 2023 to May 2024

Therapeutic approach	Number of patients
Lifestyle changes	30
Psychological therapy	22
Only community-based psychotherapy	1
Only psychotherapy at our centre	11
Combined centre- and community-based care	10
Community-based physical therapy	18
Scheduled analgesic therapy	10

These included tailored recommendations on sleep hygiene, activity pacing, return-to-school strategies, graded physical activity, and self-care (e.g., hobbies, balanced diet, hydration, social interaction), alongside support for establishing consistent routines and reducing pain-avoidance behaviours. Psychological support was offered to most patients (*n* = 21); except for one case managed locally with supportive therapy, all received structured interventions such as CBT, relaxation training, EMDR, and/or clinical hypnosis. EMDR was used in five patients: three with procedural hyperalgesia and anxiety (one also receiving systemic family therapy and pre-procedural hypnosis for lumbar puncture), one with CRPS, and one with sickle cell disease. Hypnosis was used in three cases: one with phantom limb pain and two with sickle cell disease (one combined with EMDR). In ten patients, centre-based psychological care was integrated with local professionals trained in paediatric pain management to ensure continuity, accessibility of care and long-term engagement. Physiotherapy was prescribed in 18 cases, focusing on functional restoration and movement re-education, with all referred to trusted community services. The average time from referral to activation of local services was around one month. In contrast, scheduled analgesic therapy was the least frequently adopted therapeutic approach (Table 2).

Ten patients (median age: 13 years, range: 2–17 years) received scheduled analgesic therapy due to persistent, severe, or functionally impairing pain. In this subgroup, underlying conditions included osteonecrosis associated with sickle cell disease (*n* = 3), recurrent headaches in sickle cell disease (*n* = 1), phantom limb pain (*n* = 1), musculoskeletal pain in patients with cerebral palsy, neuromuscular disease, or post-abdominal surgery (*n* = 3 in total), vulvodynia (*n* = 1) and neurofibromatosis type 1 (*n* = 1). In this subgroup, underlying conditions included osteonecrosis associated with sickle cell disease (*n* = 3), recurrent headaches in sickle cell disease (*n* = 1), phantom limb pain (*n* = 1), musculoskeletal pain in patients with cerebral palsy, neuromuscular disease, or post-abdominal surgery (*n* = 3 in total), vulvodynia (*n* = 1) and neurofibromatosis type 1 (*n* = 1). Pain phenotypes of these patients were classified as nociceptive (*n* = 3), neuropathic (*n* = 4), mixed nociceptive/neuropathic (*n* = 2), and nociplastic (*n* = 1). The most frequently prescribed agents were gabapentin (*n* = 7), followed by opioids (oral morphine or oxycodone, *n* = 3), NSAIDs (*n* = 2), amitriptyline (*n* = 1) and alprazolam (*n* = 1). One patient with a prominent emotional component was also managed by a child neuropsychiatrist, who initiated tailored psychopharmacological treatment. Opioid therapy was optimized in two patients: one transitioned from transdermal fentanyl to oral morphine, and another from morphine to oxycodone. Scheduled analgesic therapy was well tolerated, except in one case in which gabapentin was discontinued due to excessive daytime somnolence and lack of efficacy.

Patients’ pain experiences over time was monitored using pain diaries, typically completed for at least 3–4 weeks after the initial visit and after any treatment adjustment, and for longer periods when clinically indicated. All therapeutic plans were shared with the referring physician and the child’s primary care provider, as well as with any other specialists involved in the child’s care. In the most complex cases, multidisciplinary meetings were held to discuss and coordinate management strategies. The primary care provider played a key role in monitoring potential drug-related side effects and ensuring adherence to the proposed treatment plan. Only one patient with sickle cell disease required hospital admissions for episodes of acute chest syndrome. No other patients required hospitalisation or escalation to urgent care due to pain-related episodes.

Our multimodal therapeutic approach resulted in a ≥ 50% reduction in baseline pain intensity over time in all patients, with the exception of the aforementioned drop-out case. The median time to achieve this reduction was 4 months (IQR 2.0–6.25). Pain improvement was associated with reduced use of breakthrough medication, better sleep quality, improved mood, greater participation in daily activities, and decreased school absenteeism.

## Discussion

This study describes the feasibility of a structured, interdisciplinary outpatient paediatric specialised pain service at the University of Padua. Despite the modest cohort size, our experience suggests that such a multimodal care pathway can be successfully implemented across care levels, ensuring continuity and accessibility of care, while yielding a positive impact for children and adolescents with chronic and complex pain arising from diverse underlying conditions. It is well established that inadequate pain management leads to significant consequences for the overall well-being of both the child and their family [1, 2]. The interdisciplinary clinical model adopted in our clinic is an early-stage framework inspired by that advocated by Charles Berde and colleagues at Boston Children’s Hospital—one of the world’s most advanced paediatric pain programs. Their model includes an intensive interdisciplinary pain rehabilitation day program for children with chronic pain [9]. Although detailed caseload analyses have not been published, available data suggest that their Paediatric Pain Treatment Service evaluates approximately 500 patients per year and had collected data on over 2,500 cases in its pain data repository as of 2019 [10]. This contrasts with our current volume and structure. In fact, our model is organized across three levels of care: basic and specialist care at the community level, and high-complexity interventions centralized at our referral centre. The majority of patients are managed in primary and secondary care settings, with continuous supervision and clinical oversight provided by our centre reference team, while children referred to our outpatient clinic typically represent cases that have not responded to previous interventions at lower levels of care. This structure offers several advantages: patients are not hospitalised and can remain at home, where they receive high-quality, personalized support, backed by 24/7 on-call availability and the option to adjust the therapeutic plan as needed. Such an approach is designed to meet the individual needs of both the child and their family, while at the same time minimizing the impact on their daily lives. Furthermore, by avoiding unnecessary hospital admissions and promoting coordinated community-based management, this model may contribute to reducing the overall economic burden on the healthcare system.

In our cohort, the majority of patients with pain (18/28, 64%) presented with complex phenotypes characterized by mixed nociceptive/neuropathic mechanisms, nociplastic components, and prominent psychological dimensions. This distribution further supports the need to move beyond a reductionist, symptom-based approach, and instead adopt a multidimensional biopsychosocial model of assessment and intervention [4, 11]. Within this framework, it is worth noting that lifestyle modifications and psychological support represented a cornerstone of most individualised treatment plans, consistent with findings reported in the literature [12, 13]. Non-pharmacological strategies have also been shown to reduce anxiety, improve pain management skills, and enhance overall functioning, even in the absence of pharmacological interventions [12, 13].

This study has several limitations: its retrospective design, small sample size, and the lack of use of validated tools for assessing key parameters such as functional status and quality of life. Future research should prospectively follow larger cohorts, integrating patient-reported outcome measures such as the Functional Disability Inventory and the Paediatric Quality of Life Inventory, as well as outcomes like school attendance and psychological well-being. Long-term follow-up of discharged patients would provide insight into pain recurrence, new complaints, and overall recovery, allowing a comprehensive evaluation of our interdisciplinary approach.

## Conclusion

This study provides evidence supporting the feasibility and clinical value of interdisciplinary paediatric pain services. Given the significant prevalence of chronic pain in children, there is a strong rationale to implement and scale up such models across the Italian healthcare system. The success of these programs relies on the availability of interdisciplinary expertise and institutional investment in training and integrated care delivery. While complete pain resolution may not always be achievable for all patients, promoting long-term self-management and family-based strategies should remain a central therapeutic goal.

## Data Availability

No datasets were generated or analysed during the current study.

## Abbreviations

IASP: International Association for the Study of Pain
NSAIDs: Non-Steroidal Anti-Inflammatory Drugs
CBT: Cognitive Behavioural Therapy
EMDR: Eye Movement Desensitization and Reprocessing
CRPS: Complex Regional Pain Syndrome
