# Correction to: Interdisciplinary management of paediatric chronic and complex pain: the experience of a specialised outpatient clinic in Italy

**DOI:** 10.1007/s00431-025-06493-y

**Published:** 2025-09-26

**Authors:** Annalisa Salerno, Francesca Rusalen, Valentina De Tommasi, Mariangela Rosa, Irene Maghini, Grazia Ghiraldo, Franca Benini

**Affiliations:** 1 Department of Women’s and Children’s Health, University of Padua, Padua, Italy; 2 Paediatric Pain and Palliative Care Service, Department of Women’s and Children’s Health, University Hospital of Padua, Padua, Italy

**Correction to: European Journal of Pediatrics (2025) 184:608**

10.1007/s00431-025-06451-8

The authors regret that an error occurred in the recently published article, "Interdisciplinary management of paediatric chronic and complex pain: the experience of a specialised outpatient clinic in Italy," in the European Journal of Pediatrics.

In the published version, the sentence currently reads: “Nine patients (median age: 13 years, range: 2–17 years) received scheduled analgesic therapy due to persistent, severe, or functionally impairing pain.”

This is incorrect. The correct number of patients is ten, which is consistent with the subsequent list of conditions provided in the article. This error occurred due to a misinterpretation during the proof stage.

The corrected sentence should read: “Ten patients (median age: 13 years, range: 2–17 years) received scheduled analgesic therapy due to persistent, severe, or functionally impairing pain. In this subgroup, underlying conditions included osteonecrosis associated with sickle cell disease (n = 3), recurrent headaches in sickle cell disease (n = 1), phantom limb pain (n = 1), musculoskeletal pain in patients with cerebral palsy, neuromuscular disease, or post-abdominal surgery (n = 3 in total), vulvodynia (n = 1) and neurofibromatosis type 1 (n = 1).”

The authors apologize for any confusion this error may have caused.

The original article has been corrected.

